# Human Hair, Baltic Grey Seal (*Halichoerus grypus*) Fur and Herring Gull (*Larus argentatus*) Feathers as Accumulators of Bisphenol A and Alkylphenols

**DOI:** 10.1007/s00244-017-0402-0

**Published:** 2017-04-27

**Authors:** Iga Nehring, Marta Staniszewska, Lucyna Falkowska

**Affiliations:** Institute of Oceanography, University of Gdansk, Al. Marszałka Piłsudskiego 46, 81-378 Gdynia, Poland

## Abstract

The purpose of the study was to determine the concentration of bisphenol A (BPA), 4-*tert*-octylphenol (OP), and 4-nonylphenol (NP), in human hair, the fur of Baltic grey seals and the feathers of herring gulls. Hair was collected from 42 volunteers, while grey seal fur (*n* = 17) came from the seal centre in Hel (Marine Station of Institute of Oceanography, University of Gdansk) and gull covert feathers (*n* = 26) were collected from dead herring gulls along the Southern Baltic coast. Assays of phenol derivatives were conducted using the high performance liquid chromatography with fluorescence detection technique. In human hair, the mean BPA concentration amounted to 411.2 ng g^−1^ dw, OP 131.2 ng g^−1^ dw, NP 4478.4 ng g^−1^ dw, in seal fur BPA 67.5 ng g^−1^ dw, OP 62.8 ng g^−1^ dw, NP 39.1 ng g^−1^ dw, and in feathers BPA 145.1 ng g^−1^ dw, OP 162.0 ng g^−1^ dw, NP 37.7 ng g^−1^ dw. The increase of the analysed EDCs in hair was significantly influenced by diet rich in products of marine origin, as well as hair colouring, heating up food in plastic containers, using home cleaning products without protective gloves and wearing newly purchased clothes without washing them first. The concentration of phenol derivatives in seal fur was influenced solely by the uniform diet rich in fish. In birds, the feeding area during molting significantly influenced the concentration of BPA, OP and NP found in covert feathers.

**Electronic supplementary material:**

The online version of this article (doi:10.1007/s00244-017-0402-0) contains supplementary material, which is available to authorized users.

Bisphenol A (BPA), 4-*tert*-octylphenol (OP) and 4-nonylphenol (NP) belong to the group of Endocrine Disrupting Compounds—EDCs, as they are capable of disrupting homeostasis, embryonic development and reproduction (EPA 2010).

In Europe, 100 thousand tonnes of alkylphenols and 5.2 million tonnes of BPA are produced yearly, which constitutes 1/3 of global production (EA 2005). According to the decision of the European Commission, since March 2011 it has been forbidden to use bottles containing BPA for feeding babies and to introduce into the market products containing more than 0.1% nonylphenol (European Commision 2009). Still, the use and handling of these EDCs within the EU is considerable. BPA is found in various everyday objects (e.g. thermal paper receipts, bike helmets, police shields, reading glasses, circuit boards, flat screen televisions and smart phones), in items which have contact with food (e.g. cans, plastic containers) and in car and home equipment, as well as in medical products (e.g. incubators, nebulisers, implants, artificial joints, internal electronics) (ECHA 2008, American Chemistry Council 2016). Particularly 4-Nonylphenol is used mainly during the production of as nonionic surfactants in detergents, cleaners, industrial applications, but also in cosmetics. In Europe there are no bans or limitations on the usage of OP. 4-*tert*-Octylphenol is found in, among other things, car tyres, paints and varnishes (HELCOM 2010). The widespread usage of these compounds results in their measurable presence in the environment of the Southern Baltic. A wide range of BPA, OP and NP concentrations was found in the surface water, between <1.0 and 834.5 ng dm^−3^, sediments (<0.08–249.08 ng g^−1^ dw) in plankton (<0.8–769.2 ng g^−1^ dw) and in fish (<0.8–798.4 ng g^−1^), which are a source of food for seabirds, seals and humans (HELCOM 2010; Koniecko et al. 2014; Staniszewska et al. 2014, 2015a, b, 2016a, b).

For organisms, the dietary intake is the main route of exposure to alkylphenols and bisphenol A (EC 2002, Xiao et al. 2006, American Chemistry Council 2016). Predatory birds and mammals, occupying the highest trophic level, are the most exposed to the accumulation of hydrophobic organic compounds (Jaspers 2008). In humans, an additional risk is the migration of substances from plastic containers into food or cosmetics contained within (Touze-Foltz et al. 2012). Other routes of exposure to EDCs are inhaling polluted air (Berkner et al. 2004; Fu and Kawamura 2010), or maternal transfer (Chen et al. 2008).

In the process of evolution, organisms have developed a number of methods for detoxication and removal of various chemical compounds. One way of eliminating toxic substances is to transfer them from the parents to their offspring, also by laying eggs (Grajewska et al. 2015). Xenobiotic elimination is also achieved by incorporating toxic substances into feathers, claws, nails and hair, as well as removing them with excrement (Covaci and Schepens 2001; Kintz 2007; Schramm 2008).

Feathers are frequently used as an indicator of environmental pollution with organic compounds (Dauwe et al. 2005; Van den Steen et al. 2007; García-Fernández et al. 2013). As opposed to human hair, which grows constantly, feathers are formed in a few weeks during molting (Jaspers et al. 2006). Xenobiotics incorporated into feathers are chemically and physically stable (Thompson et al. 1998; García-Fernández et al. 2013). These biotic matrices can be collected in a non-invasive way that is safe for the organism (Nakao et al. 2002, 2005). Hair and fur samples are often used in epidemiology and ecology studies.

Compared with forensic and clinical fields, the literature dealing with human hair analysis for the detection of organic pollutants is rather poor. Available publications report only a few substances belonging to the groups of organochlorine pesticides (OCP), polybrominated diphenyl ethers (PBDEs), polycyclic aromatic hydrocarbons (PAHs), polychlorinated biphenyls (PCBs) and dioxins (PCDDs) (Schramm et al. 1992; Tsatsakis et al. 2008; Covaci and Schepens 2001; Kintz 2007). There is no available data on the presence of bisphenol A and alkylphenols in seal fur, bird feathers and the hair of Polish inhabitants. The hair of Poles has been used before for the assays of a number persistent organic pollutants (POPs), such as (PCBs), pesticides (DDT), hexachlorocyclohexanes (HCHs), or perfluorinated compounds (Król et al. 2014). Both POPs (PCB, DDT, HCH) and mercury (i.e., organic mercury, MeHg) were found in the feathers of birds inhabiting the Baltic area. Studies showed that feathers are depot tissues for the accumulation of these compounds (Behrooz et al. 2009; Szumiło-Pilarska et al. 2016).

The fact that there are no unequivocal bans for the production or use of EDCs in many products that humans come into everyday contact with indicates that the inhabitants of Pomerania (a region of Poland situated on the Southern Baltic), just like people from other parts of Europe, are exposed to such compounds.

The purpose of the present study was determine concentration of BPA and alkylphenols in human hair, grey seal fur (*Halichoerus grypus*) and herring gull (*Larus argentatus*) feather. Authors also attempted to indicate the potential factors BPA, OP, NP in herring gull feathers, grey seal fur, and in the hair of inhabitants of Pomerania.

Exposure to the endocrine active phenol derivatives differs in organisms on different trophic levels. Organisms from a lower trophic level are exposed to the EDCs mainly by the penetration of water or sediment. Birds and marine mammals that are at the top of the marine food web are potentially more exposed to penetration of xenobiotics. Pollution constantly circulate in the environment and man is constantly exposed to their action Therefore, it is essential to identify ways of exposure and the possibility of intake of endocrine active compounds for the human body. Another rationale for choosing people living in the coastal zone of the Gulf of Gdansk is the incidence rates of cancer and thyroid dysfunction associated to the high exposure of EDCs in Pomerania, as reported by Ministry of Health. Two types of cancer—breast and groin lymph—are responsible of causing 13.4% of deaths among women and 8% of deaths among men (Ministry of Health 2015).

We hypothesize that synthetic materials, home-cleaning products, and cosmetics containing EDCs, present in the human environment, may penetrate the human skin and have effects that could be comparable to alimentary exposure.

## Materials and Methods

### Species Characterisation

The herring gull (*L. argentatus*) is a predominant species in the Gulf of Gdansk area outside the breeding season (Meissner and Rydzkowski 2007). Communal landfills are a significant source of food for these gulls and they also accompany fishing boats in large numbers at the time of fishing, which provides them with easy access to fish and entrails left after gutting. Diet depend on accessibility of food, including marine fish and invertebrates, small amphibians, annelids, eggs and nestlings of other birds, carrion, and waste found in urban environment. Municipal waste create very important food source for gulls (Meissner and Batleja 2007). Gulls wintering in the area of Gulf of Gdansk are frequently feeding in three large municipal waste dumps, in Gdansk Szadółki, Łężyce near Gdynia, and in vicinity of Władysławowo.

Gulls search for food in many different areas, travelling over large distances—where these seabirds can be expose to contaminants—and thus indicates the contamination of their habitat. Bird feathers are collected easily and noninvasively and are considered by many researchers to be good indicators of dietary exposure (Cotín Martínez 2012; Falkowska et al. 2013).

The grey seal (*H. grypus*) is one of the most important mammal species found in the Baltic Sea. The animals live close to the coast and form small groups, feeding mainly on herring, sprat, salmonid fish, and invertebrates grey seals target and capture their food exclusively in water, both in the surface zones and at depths reaching 20–30 m (Lundström et al. 2010).

The Polish region of Pomerania, situated on the Southern Baltic, is inhabited by nearly 2.5 million people. The area features agriculture, many sectors of industry connected with the sea, as well as fisheries and tourism. In comparison with the population of other Polish regions, the inhabitants of Pomerania are within the group of people predispose to a higher risk of cancer. (Bartosińska et al. 2005). Doctors in Poland place great importance on the incidence of diseases connected with under- or overactive thyroid hormone, which affects 22% of Poles, mainly women (Ponichtera and Borowiak 2008).

### Sampling

Feathers used in the studies were collected from dead herring gulls *L. argentatus* (*n* = 26) inhabiting Gdynia harbour, the Vistula estuary, and the fishing port in Władysławo over the period 2010–2012. The causes of death of birds have not been studied. One male herring gull was characterized by a 10% of the state debilitating, possibly due to peritonitis (Falkowska 2016).

Among the birds there were 13 females and 13 males, and covert feathers were selected from both juvenile gulls (*n* = 10) and mature specimens (*n* = 16). The age of birds was determined on the basis of plumage (Olsen and Larsson 2004). Birds in their first winter plumage were categorized as juvenile, and birds in their fourth and final plumage were considered to be mature. Because of sexual immaturity, the gender of some birds was determined in genetic studies through DNA amplification using the PCR method (Fridolfsson and Ellegren 1999).

Fur samples were collected from grey seals at the Marine Station of the Institute of Oceanography, University of Gdansk. The seal centre has four females (aged 22–26 years) and two males (10 and 29 years old) living there permanently. The Marine Station works on the reintroduction of the grey seal into the Baltic and every year between one and four seal pups are born there. Fur samples of mature females (*n* = 5) that pupped in the years 2014–2015 and their pups (*n* = 12) were collected using metal scissors or, in the case of short fur, were shaved off.

Hair samples were collected from anonymous donors: adults aged 25–55 years (*n* = 15), and from school students aged 16–17 years (*n* = 27). The hair was collected from the back of the head using stainless steel scissors, which were cleaned with acetone after each sample collection. The volunteers who have responded anonymously to a questionnaire concerning age, sex, diet (the main problem: the amount of fresh fish, processed fish, and sea products or diet devoided of fish), heating food in plastic containers, paying attention to the signs on the plastic packaging, the use of cosmetics and household chemicals (hair dye, use biocosmetics, or use protective gloves during using surfactants), and the habit of washing newly purchased clothes. A questionnaire facilitating the identification of potential factors affecting on the exposure of organisms to the phenol derivatives also was included in the survey.

During collection, storage, and all further stages of analysis, great care was taken to avoid contact between the samples and synthetic materials. At the laboratory, the samples were washed in acetone to remove any soiling present on the surface of the hair/feathers/fur and then dried (Thompson et al. 1998; Jaspers et al. 2008, 2011).

### Determination of BPA, OP, and NP

All the reagents (water, acetonitrile, and methanol) by (Merck Germany) were HPLC pure. Chloric acid and ammonium acetate were by POCh. Standards of bisphenol A, 4-*tert*-octylphenol, and 4-nonylphenol by (SIGMA-ALDRICH^®^ USA) were of high purity (>97%). Standards for the preparation of a calibration curve, in the following concentrations (10, 25, 50, 75, 100 ng cm^−3^) were prepared in methanol.

To quantify BPA, OP, and NP, 0.1-g samples (hair, fur, feathers) were taken and extracted with mixture of 8 cm^3^ methanol, 2 cm^3^ of 0.01 mol dm^−3^ ammonium acetate, and 100 µcm^3^ chloric acid (VII) in an ultrasonic bath (10 min, 20 °C). The supernatant after centrifugation was supplemented to a volume of 20 cm^3^ by of 0.01 mol dm^−3^ ammonium acetate. Next, they were purified on Oasis HLB glass cartridges (5 ml/200 mg) (waters) according to the method set out by Xiao et al. (2006) and Staniszewska et al. (2014, 2015b). After the enrichment step, BPA, OP, and NP were eluted from the columns with methanol.

The obtained extracts were evaporated until dry and supplemented with 200 µl of acetonitrile. Chromatographic analysis using a liquid chromatograph with a fluorescence detector was performed on a HYPERSIL GOLD C18 PAH column (Thermo Scientific) (250 × 4.6 mm; 5 µm) with a mobile phase (acetonitrile and water) in gradient conditions. Glass vessels were used, suitably prepared by etching with nitric acid at a concentration of 3.5 mol dm^−3^ for 24 h and drying at 200 °C.

The amounts of BPA, OP, and NP recovered, determined through a quintuple analysis of each kind of sample containing a known amount of the standard, were in average 85% (BPA), 87% (OP), and 94% (NP). Relative standard deviation was below 10%.

The limit of quantification (LOQ) of the method was determined as a tenfold signal to noise ratio for a sample with very low analyte content and amounted to: 2.0 (BPA), 0.5 (OP), 0.5 ng g^−1^ sm (NP). The background (lab procedural blanks) was checked every time a new batch of columns was used. The obtained “background” values for BPA, NP, and OP were <LOQ.

### Isotope Analysis

Stable nitrogen (δ^15^N) and carbon (δ^13^C) isotopes were used as ecological tracers of seabirds trophic level and source of carbon. The analysis was performed in gull muscles using a Sercon 20–22 Continuous Flow Isotope Ratio Mass Spectrometer (CF-IRMS) coupled with Sercon SL elemental analyser for simultaneous carbon–nitrogen–sulfur (NCS). Results were expressed as differences in isotopic ratios as parts per thousand (‰) according to the formula:

$$\delta X = \left[ {\left( {R_{\text{sample}} /R_{\text{standard}} } \right)^{ - 1} } \right] \times 1000$$

where *X* represents ^15^N or ^13^C and *R* is the corresponding ^15^N/^14^N or ^13^C/^12^C ratio. The standard for ^15^N was atmospheric nitrogen (AIR) and for ^13^C Pee Dee Belemnite (PDB) was used as a reference.

### Statistical Analysis

The statistical analysis of the obtained results was performed using the STATISTICA 10 programme by Stat Soft. The normality of data distribution was tested with the Shapiro–Wilk test (*p* < 0.05), and it was demonstrated that the distributions deviated from normal, so the Kruskal–Wallis test was used to determine the significance of differences (*p* < 0.05). Correlation coefficients were determined using the Spearman coefficient, adopting a 95% confidence interval.

## Results

Bisphenol A, 4-*tert*-octylphenol, and 4-nonylphenol were found in 98% of feather, hair, and fur samples. The lowest BPA concentrations were in seal fur (67.5 ng g^−1^ dw), whereas the highest were detected in human hair and were nearly six times higher of that measured in seal fur and three times higher than the concentration in gull feathers (145.1 ng g^−1^ dw). Hair samples were characterised by the decisively highest mean NP concentrations, which were as much as 100 times higher than those detected in fur or feathers (Table 1). In the three studied matrices, OP concentrations were the least diversified.

**Table 1:** Concentrations of bisphenol A (BPA), 4-*tert*-octylphenol (OP), and 4-nonylphenol (NP) in samples of herring gull feathers, Baltic grey seal fur, and the hair of Pomeranian inhabitants

Sample type	BPA (ng g^−1^ dw)	OP (ng g^−1^ dw)	NP (ng g^−1^ dw)
Feathers *n* = 26			
Md	103.7	124.3	29.4
*x* ± SD	145.1 ± 113.6	162.0 ± 122.1	37.7 ± 35.0
Min–max	29.3–512.4	28.4–563.6	4.9–151.3
Fur *n* = 17			
Md	70.2	71.8	24.7
*x* ± SD	67.5 ± 36.5	62.8 ± 20.7	39.1 ± 27.2
Min–max	<LOQ—137.2	12.7–86.5	5.5–91.3
Hair *n* = 42			
Md	337.5	109.9	289.0
*x* ± SD	411.2 ± 365.5	131.2 ± 103.6	4478.4 ± 5279.5
Min–max	26.1–1498.6	5.6–481.5	5.4–23,829.5

*Md* median value, *x* mean value, *SD* standard deviation, *min*–*max* range of concentration, *LOQ* limit of quantification, *dw* dry weight

### Feathers

In the herring gull feathers, the mean OP concentration (162.0 ng g^−1^ dw), was mor ethan four times higher than of that measured for NP. NP also occurred in the widest range of concentrations. BPA concentrations, however, were similar to OP concentrations. BPA concentrations in covert feathers of a mature female collected in spring from Gdynia harbour were relatively higher than the concentration in feathers of Gull found during the end of summer and in winter at the fishing port of Władysławowo and at the Vistula estuary. In the feathers of gulls from the two habitats, BPA concentrations were nearly fourfold lower, whereas NP concentrations were 14-fold lower. Neither age nor gender had any influence on the diversification of EDC concentrations in feathers, as confirmed by a Kruskal–Wallis test [BPA: KW-H(1;25) = 0.145; *p* = 0.7034; OP: KW-H(1;22) = 0.0011; *p* = 0.9738; NP: KW-H(1;24) = 0.084; *p* = 0.7720].

### Fur

Mean concentration of BPA, OP, and NP in the fur of mature grey seals were similar (i.e., 62.1; 65.3; 23.8 ng g^−1^ dw respectively) to those in the fur of seal pups (i.e., 68.8; 62.0; 43.8 ng g^−1^ dw respectively). The differences in EDC concentration in relation to age were not statistically significant [BPA: KW-H(1;16) = 0.0588; *p* = 0.8084; OP: KW-H(1;16) = 0.0588; *p* = 0.8084; NP: KW-H(1;16) = 0.7206; *p* = 0.3960].

### Hair

The questionnaire on diet and lifestyle was completed by everyone in the study group (15 adults and 27 teenagers). Responses to the questionnaire by surveyed volunteers are included in Figure 1 (Supplementary material). All of these activities could have an influence on the presence and level of EDC concentrations in hair. No statistically significant differences were observed between men and women, regardless of their age [BPA: KW-H(1;42) = 0.3324; *p* = 0.5642; OP: KW-H(1;42) = 3.2175; *p* = 0.0729; NP: KW-H(1;42) = 1.0506; *p* = 0.3054].

Only 14% of the respondents declared that they did not consume food of marine origin (fish, seafood). All others included such products in their diet. Seventy-nine percent of them consumed fresh and frozen fish and seafood, of whom 51% also consumed processed fish (smoked fish, canned fish). Only 7% of the respondents stated that they did not eat fresh fish but only processed. More than 60% of respondents consumed marine food more than once per month. One quarter of the respondents heated up food in plastic containers. Only one person responded positively to questions about paying attention to the plastic identification symbols found on, e.g. mineral water bottles. One-third of respondents used natural cosmetics, potentially free from EDCs. Only 29% of the respondents used protective gloves, and 33% washed new clothes before wearing. But only 9.5% of them protected themselves by both washing new clothes and using gloves. Twenty-six percent of respondents used hair dyes, of whom 36% also heated up food in plastic containers (Figure 1, supplementary material). There were differences between NP concentrations found in the hair of people who heated up food and the hair of those who did not. That was confirmed by a Kruskal–Wallis test [KW-H(2;37) = 10.0083; *p* = 0.0067].

The highest NP concentration (23,829.5 ng g^−1^ dw) was found in the hair of a 26-year-old female, whereas the lowest was in the hair of a 17-year-old female (5.4 ng g^−1^ dw). Both dyed their hair and had similar lifestyles. The difference among females was that the female with low concentration washed her new clothes before wearing them.

The highest OP values (481.5 ng g^−1^ dw) were found in the hair of a 17-year-old female, who followed a gluten-free diet and consumed only fresh fish. She bought natural cosmetics and washed her newly purchased clothes but did not wear protective gloves when using surfactants. A low OP concentration (5.6 ng g^−1^ dw) was found in the hair of a 26-year-old male, who declared that he did not wash his newly purchased clothes or wear protective gloves when using surfactants and that his diet contained both fresh and processed sea fish.

An elevated BPA concentration (1498.6 ng g^−1^ dw) occurred in the hair of a 17-year-old female student and a low concentration (26.1 ng g^−1^ dw) in a 16-year-old. Neither of them dyed their hair, but the first female student did not wear protective gloves, wash new clothes, or use natural cosmetics. The other student had a habit of washing new clothes but did not use protective gloves and heated up food in plastic containers.

## Discussion

Endocrine disrupting compounds can enter into the system via three routes: inhalatory, alimentary, and through the skin. The concentrations of BPA, OP, and NP in gull feathers, grey seal fur, and human hair indicated some differences in the level of the organisms’ exposure to these compounds (Table 1). The concentrations of phenol derivatives measured in hair were found to be up to an order of magnitude higher than in gull feathers or seal fur. Because OP concentrations in all matrices were the least abundant and because of the biotransformation of contaminants (e.g. enzymatic metabolizm), humans have a greater metabolic capacity to remove NP or BPA.

### BPA, OP, and NP in Gull Feathers

Herring gulls are omnivorous. In the coastal zone, in their search for fish and seafood, they often accompany fishing boats during fishing. When consuming fish, seabirds consume nutritious food that is richer in BPA than alkylphenols. This is confirmed by previous studies, in which the mean BPA concentration in the muscles of the Baltic herring was the highest (98.6 ng g^−1^ dw) in relation to alkylphenols (OP < 0.8 ng g^−1^ dw, NP 9.8 ng g^−1^) (Staniszewska et al. 2014). In a mature female from the breeding colony at Gdynia harbour, the highest BPA concentrations occurred after the breeding period, end of spring/beginning of summer. While feeding, gulls make an effort to obtain the most nutritious food, as confirmed by bird pellets (fish bones and other fish parts) found in their nesting place. Such foraging behaviour of seabirds also is reported by Forero & Hobson (2003). The fact that the prey of gulls from Gdynia harbour came from higher trophic levels was confirmed by the results for the stable nitrogen isotope (11.79–12.03‰ δ^15^N) compared with gulls from the other two colonies: in Władysławowo (8.77–10.97‰ δ^15^N) and the Vistula estuary (8.10–9.49‰ δ^15^N). Moreover, gulls nesting in Gdynia during breeding obtained their food from a smaller area (−22.89 to −22.92‰, δ^13^C) compared with nesting in Władysławowo (−23.37 to −25.67‰ δ^13^C) and at the Vistula estuary (−22.89 to −24.37‰ δ^13^C) during the late summer and autumn.

The fact that the trophic level of gull food was lowered in late summer and autumn was confirmed by stable nitrogen isotopes δ^15^N, indicating diet items of anthropogenic origin likely to be found in communal landfills. When feeding in such places, herring gulls swallow pieces of accompanying material, such as foil or plastic, a fact reported by ornithologists who find pieces of synthetic materials (micro plastics or microbeads) in pellets. Phenol derivatives entered the food is eventually accumulated in the feathers. This process occurs effectively due to the properties of keratin to biosorption (physiosorption and chemisorption) (Manshouri et al. 2012; Atikah 2013). Keratin, one of the proteins that form a feather, involves phenol derivatives (Collie and Ghosh 2014). Feathers are created for several weeks or longer and have a close relationship with the quality and availability of food (Verbeek 1977).

The age of a specimen as a factor influencing the level of EDC concentrations in feathers is suggested by the literature (Falkowska et al. 2013; Szumiło-Pilarska et al. 2016). OP was the only compound for which the Kruskal–Wallis test indicated differences between concentrations in the herring gull feathers collected from juvenile specimens (on average 109.9 ng g^−1^ dw; *n* = 10) and mature ones [202.2 ng g^−1^ dw; *n* = 16: KW-H(1;26) = 1.7361; *p* = 0.1876; OP: KW-H(1;23) = 4.1885; *p* = 0.0407; NP: KW-H(1;25) = 0.3394; *p* = 0.5602]. Despite the lack of statistically significant differences, higher mean BPA concentrations were measured in the feathers of mature birds (153.3 ng g^−1^ dw) compared with the feathers of juvenile ones (132.0 ng g^−1^ dw). The opposite correlation was indicated in the case of NP; young birds had a concentration of 48.3 ng g^−1^ dw and mature ones 30.7 ng g^−1^ dw. Because the molting of gulls found in the coastal zone of the Baltic often takes place during or after the breeding season, mature gulls could have been more contaminated with BPA than the juvenile birds.

Gender did not have an influence on the concentration of phenol derivatives [BPA: KW-H(1;25) = 0.145; *p* = 0.7034; OP: KW-H(1;22) = 0.0011; *p* = 0.9738; NP: KW-(1;24) = 0.084; *p* = 0.7720]. It is likely, however, that bird gender can influence concentration patterns of the studied EDCs, as indicated by the proportions of the particular compound concentrations in the feathers of mature gulls. For BPA the ratio was 1.2, 0.9 for OP, and 0.8 for NP. Higher BPA concentrations in the feathers of males than in the feathers of females can be attributed to egg laying and the period of feeding nestlings by both parents. Another possibility is that there is inequality between the efficiency of EDC removal into eggs and into feathers. These two processes of contaminant elimination from a female body can be a effective way of contaminant elimination (e.g. maternal transfer), as confirmed by studies on birds in the breeding and non-breeding seasons (Bond and Diamond 2009; Szumiło-Pilarska et al. 2016). It also is suggested by the results of studies investigating chlorinated organic compounds and mercury in the tissues and organs of penguins (Falkowska et al. 2013).

### In Seal Fur

The size of the EDC load introduced into a seal’s body was determined by a uniform diet composed mainly of herring. Comparing the results for phenol derivative concentrations in seals’ diet (whole herring BPA 105.8 ng g^−1^ dw; OP 19.0 ng g^−1^ dw; NP 24.0 ng g^−1^) (Staniszewska et al. 2014) and in the fur of seals from the Hel seal centre (Table 1), it follows that in fur the accumulation of OP and NP was more effective than that of BPA. On the other hand, the removal of BPA from the system in seals was more effective through defecation (Genuis et al. 2011). This was indicated by the particularly high values that BPA reached in feaces (mean value 7695.6 ng g^−1^ dw), which were as much as 200 times higher than OP and NP concentrations (personal observation). Phenol derivatives also are removed from the body in urine (Genuis et al. 2011). On that basis, it can be suggested that alkylphenols accumulate better in animal tissues and organs than BPA does.

Marine mammals, feeding mainly on fish, also can be exposed to EDCs by adsorbing such compounds from the water. This problem, however, has not been discussed in literature. Koniecko et al. (2014) and Staniszewska et al. (2015b) indicated that phenol derivative concentrations increased in the Gulf of Gdansk in summer with the arrival of tourists seeking seaside recreation. At that time, the use of products containing BPA, OP, and NP increased and so did passenger ship traffic (4-nonylphenol is present in petroleum) (European Commission, 2002). The air appears to be of rather low significance as a route of exposure of phenol derivatives into seal systems. Studies thus far have shown that in the air over Gdynia, the mean BPA, OP, and NP concentrations in small aerosol fractions PM < 2.5 were within the range of 0.1–1.2 ng m^−3^ (Lewandowska et al. 2012).

### In Human Hair

Phenol derivatives can penetrate into the human body mainly via the dietary route and through the skin. This was taken into account in the questionnaire addressed to the volunteers. The respiratory route was not included in the study.

Answers to the survey questions (supplementary material) indicated the correlation between EDC concentrations in human hair and the use of cosmetics and everyday items made of synthetic materials. The most significant factor that could have influenced the levels of BPA, OP, and NP in hair was the use of dyes. To a lesser extent, they were determined by the contents of cosmetics used, as well as not wearing protective gloves while using surfactants, and not washing newly purchased clothes before wearing.

Hair colouring affected NP concentrations the most. People who used hair dyes and bleaches had NP concentrations that were 50% higher (3354.7 ng g^−1^) than those who did not dye their hair. Researchers report that hair colour may limit the use of hair as a biomarker of exposure to pollutants in humans. When studying rats, Green and Wilson (1996) found that animals with black fur were characterised by higher concentrations of pharmaceuticals than animals with white fur. Similar tests were performed on hair (Rothe et al. 1997). These studies showed that people with grey hair were characterised by relatively low mean concentration of BPA (141.2 ng g^−1^ dw) and NP (90.3 ng g^−1^ dw) compared with people with other hair colour (424.7 and 2364.9 ng g^−1^ dw respectively for BPA and NP). Moreover, the products left on the scalp during colouring also could have influenced the penetration of BPA, OP, and NP into the system (EC, 2002).

Gender may influence the differentiation between concentrations in human hair, although there were no statistically significant differences found in the present study. However, NP was found in a much wider range of concentrations in the hair of females. This is connected to the fact that more women than men dye their hair. Furthermore, women use more cosmetics potentially containing nonylphenol, and they more frequently buy clothes, the production of which involves the use of this compound (EA 2013). Washing newly purchased clothes reduces the transport of endocrine disrupting compounds through the skin (Moody et al. 2010). NP introduced through the skin can be deposited in hair or nails as a result of elimination from the body. A similar exposure pathway can be caused by not using protective gloves; as many as 71% of respondents admitted to not wearing them.

Phenol derivatives present in food packaging can be transferred into the body through ingestion of food. The process of EDC partitioning into food when it is heated up in plastic containers was confirmed by Pacific Northwest Pollution Prevention Resource Center (2013). The report was preceded by a number of studies (Yang et al. 2011). The authors detected EDC release from nine food packagings when heated (the composition was not specified). Despite the fact that EDCs occur in packaging in low concentrations, what is crucial is the time that food is exposed (Muncke 2009). The concentrations of NP concentrations found in various materials ranged from <0.03 µg g^−1^ in PET bottles to 287 µg g^−1^ in foil (Fernandes et al. 2008). Nonylphenol also has been found in UHT milk cartons (40 µg kg^−1^) or HDPE milk bottles 32.3 µg kg^−1^ (Touze-Foltz et al. 2012). BPA is widely used in various packagings despite its endocrine disruption properties being known (Vom Saal et al. 2007) and also is present in internal layers of food tins (EA 2013).

Apart from EDC transfer into food from synthetic materials, the presence of these compounds in food as such also is significant. An important factor was the type of seafood consumed by the respondents. The mean EDC concentration in the hair of the people who consumed both fresh and processed fish (smoked fish, tinned fish) was higher (BPA 583.5 ng g^−1^; OP 157.0 ng g^−1^; NP 4978.5 ng g^−1^) than in the people who ate only fresh fish (339.9; 139.0; 2525.0 ng g^−1^ respectively for BPA, OP, and NP). The highest NP concentration was found in the hair of people eating only processed fish (7%), and it was four times the concentration found in people who ate only fresh fish (79% of the respondents). Additionally, more than 50% of these people had contact with hair dyes, did not pay attention to the chemical composition of the cosmetics they used, and did not wash new clothes before wearing them. All of these factors potentially increased the level of 4-nonylphenol introduced to the system and ultimately incorporated into hair.

Coefficients log*K*_*o*/*w*_ (respectively 3.4 (EC 2014) for BPA, 4.12 (US EPA 2010) for OP, and 4.48 (EA 2005) for NP) indicate the ability to accumulate in organisms studied EDCs. However as determined in previous studies (Staniszewska et al. 2014, 2015, 2016), the maximum coefficient bioaccumulation (BAF) and biomagnification (ang. Biomagnification Factor—BMF) ranged from few to few dozen and showed a small accumulation of phenol derivatives on both low (plankton, zoobenthos) and high trophic level (fish, birds), the Gulf of Gdansk. The process of accumulation is less efficient at higher trophic level and is confirmed by the high concentration of EDCs in guanie birds (Staniszewska et al. 2014). Probably at the seals or the man elimination process will be more efficient than the accumulation in tissues in medium-polluted environment.

## Summary

The study results showed that phenol derivatives in human hair, bird feathers, and seal fur were on quantifiable levels of concentration. The levels of bisphenol A and alkylphenol concentrations in these matrices, as an elimination mechanism, were influenced by dietary exposure, lifestyle, and living conditions. In each case, the alimentary route was an important source of EDC introduction, but its significance decreased in the face of the increasing number of external factors affecting the system. Humans are characterised by the large range of activities that put them in daily contact with products and packagings containing EDCs, and this was manifested by the highest concentrations in hair, particularly of bisphenol A and 4-nonylphenol. In the inhabitants of Pomerania, BPA concentrations in hair were between 26.1 and 1498.6 ng g^−1^ dw, OP from 5.6 to 481.5 ng g^−1^ dw, and NP from 5.4 to 23,829.5 ng g^−1^ dw. The diet rich in fish of the grey seal was found to be the main exposure pathway of BPA, OP, and NP, which contributed to trace values of phenol derivatives in the fur that were lower than those fund in human hair (BPA from <LOQ to 137.2 ng g^−1^ dw; OP from 12.7 to 86.5 ng g^−1^ dw; NP from 5.5 to 91.3 ng g^−1^ dw).

Herring gull feathers showed that the contamination of birds with phenol derivatives depended on the area they inhabited during molting, which also was linked to their diet (foraging in the coastal zone or on communal landfills). The influence of a diet rich in fish was manifested by elevated BPA concentrations in covert feathers of those gulls foraging in the coastal zone during the breeding period. The birds that obtained food from various trophic levels had higher concentrations of phenol derivatives in their covert feathers than seals did in their fur (BPA was found within a range of 29.3–512.4 ng g^−1^ dw; OP from 28.4 to 563.6 ng g^−1^ dw; NP from 4.9 to 151.3 ng g^−1^ dw).

Hair is a good indicator of contamination burden in the body. The hypothesis put forward at the beginning of the study was most strongly confirmed by the results for NP and moderate BPA in human hair, whereas OP concentrations did not confirm it. This study provides a basis for the formulation of further queries and exploration of this subject. Moreover, the study results should lead to promoting steps designed to protect the environment and limit exposure of humans, particularly for adolescents and young adults at the age of procreation, against routes of direct contact and introduction of phenol derivatives. A widespread public health campaign should communicate awareness about the health risks to reduce and eliminate the use of these substances.

## Electronic supplementary material

Below is the link to the electronic supplementary material.

Supplementary material 1 (DOCX 166 kb)
