# Supplementary material for: Human Hair, Baltic Grey Seal (Halichoerus grypus) Fur and Herring Gull (Larus argentatus) Feathers as Accumulators of Bisphenol A and Alkylphenols
Source: Arch Environ Contam Toxicol. 2017 Apr 27;72(4):552–61. doi: 10.1007/s00244-017-0402-0 (PMC5422498; doi:10.1007/s00244-017-0402-0)
Supplement: Supplementary file 1 — Supplementary material 1 (DOCX 166 kb) [file 244_2017_402_MOESM1_ESM.docx]

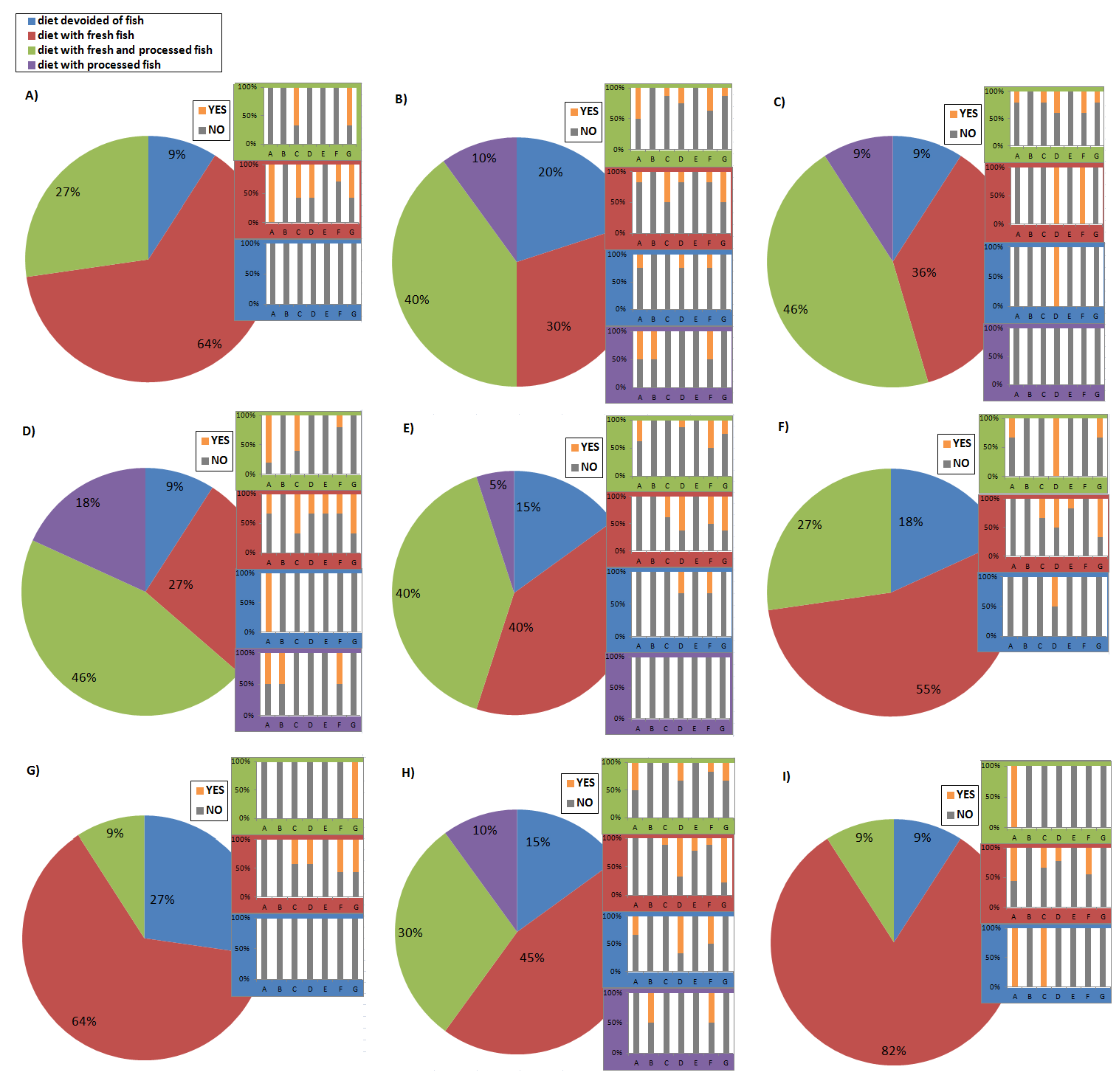


Figure 1. The percentages of respondents who did not include any fish in their diet (blue); contained only fresh fish (red), included fresh fish and fish products (green), or contained only fish products (purple) and in their hairs: concentration of bisphenol A were below the lower quartile- <106.7 ng · g^-1^ dw; B) range from lower to upper quartile: from 106.8 ng · g^-1^ d.w. to 615.5 ng · g^-1^ d.w; C) above the upper quartile: > 615.6 ng · g^-1^ d.w; concentration of 4-*tert*-octylphenol were D) <59.1 ng; g^-1^ d.w., E) from 59.2 ng· g^-1^ d.w. to 174.5 ng · g^-1^ d.w; F) > 174.6 ng · g^-1^ d.w ; concentrations of 4-nonylphenol were G) <44.5 ng · g^-1^ dw, H) from 44.6 ng ·g^-1^ dw to1534.1 ng · g^-1^ dw, I)> 1534.2 ng g ^-1^. For every range of concentration have been placed percentage of respondents who answered YES or NO on the questionnaire on: A- heating food in plastic containers, C- using hair dyes, D- using biocosmetic , Made with natural ingriedients, E-using products with sign "BPA free", F- non-use protective gloves during surfactants, G- washing newly purchased clothes
